# Growing in generosity? The effects of giving magnitude, target, and audience on the neural signature of giving in adolescence^☆^

**DOI:** 10.1016/j.dcn.2022.101084

**Published:** 2022-02-09

**Authors:** Suzanne van de Groep, Kiki Zanolie, Sarah M. Burke, Philip Brandner, Andrew J. Fuligni, Eveline A. Crone

**Affiliations:** a Erasmus SYNC Lab, The Netherlands; b Erasmus School of Social and Behavioral Sciences, Erasmus University Rotterdam, The Netherlands; c Brain and Development Research Center, The Netherlands; d Leiden Institute for Brain and Cognition, The Netherlands; e Department of Developmental and Educational Psychology, Institute of Psychology, Leiden University, The Netherlands; f Interdisciplinary Centre for Psychopathology and Emotion Regulation, Department of Psychiatry, University Medical Centre Groningen, The Netherlands; g Department of Psychiatry and Biobehavioral Sciences, University of California Los Angeles, United States

**Keywords:** Giving, Adolescence, Target, Audience, FMRI, Prosocial behavior

## Abstract

Giving is essential for forming and maintaining social relationships, which is an important developmental task for adolescents. This pre-registered fMRI study investigated behavioral and neural correlates of adolescents’ (*N* = 128, ages 9 – 19 years) small versus large size giving in different social contexts related to target (i.e., giving to a friend or unfamiliar peer) and peer presence (i.e., anonymous versus audience giving). Participants gave more in the small size than large size condition, more to friends than to unfamiliar peers, and more in the audience compared to anonymous condition. Giving very small or large amounts was associated with increased activity in the medial prefrontal cortex (mPFC) and anterior insula (AI), and older adolescents showed increased lateral and anterior PFC activation for small size giving. We observed activity in the intraparietal cortex (IPL), dorsolateral prefrontal cortex, and AI for giving to friends, but no age-related differences in this activity. Behaviorally, in contrast, we observed that older adolescents differentiated more in giving between friends and unfamiliar peers. Finally, we observed interactions between peer presence and target in the AI, and between giving magnitude and target in the precuneus. Together, findings reveal higher context-dependency of giving and more lateral PFC activity for small versus large giving in older adolescents.

## 1 Introduction

Adolescence is the period between approximately ages 8–22-years during which individuals develop mature personal and social goals, that fit the increasingly complex social world they are to navigate (Crone and Dahl, 2012). A key developmental challenge in this period is to cultivate kind and reciprocal relationships with others, with giving as one of the most important building blocks for such relationships (Crone and Dahl, 2012, Cutler and Campbell-Meiklejohn, 2019, van de Groep et al., 2020a). Prior developmental studies examined giving behavior towards unfamiliar others using the Dictator Game paradigm, in which an individual can give away valuable resources (e.g., coins or money; (Kahneman et al., 1986). These studies, that examined non-strategic costly giving, showed no behavioral differences between children, adolescents, and adults (Do et al., 2019, Gummerum et al., 2008, van de Groep et al., 2020a). Developmental differences were, however, observed in studies that utilized strategic interaction paradigms (i.e., games where giving behaviors can improve one’s own situation via reciprocity, reputation, or public good, such as the Ultimatum Game; Will and Güroğlu, 2016). Such studies in 6–13-year old children (Steinbeis et al., 2012) and 9–18-year-old adolescents (Güroğlu et al., 2009) showed that individuals give more to unfamiliar others with increasing age. Together, these studies suggest that adolescence is characterized by age-related increases in strategic motivations behind giving (Crone, 2013).

Recent evidence shows that social contextual factors also influence decision-making in adolescence. Three social contextual factors that may influence giving behavior in adolescence are giving magnitude (i.e., relative costs and benefits for self and others), the target of giving, and peer presence. First, giving has traditionally been studied using unrestricted Dictator Games, in which individuals were free to divide valuable resources as they see fit. Although most individuals make such decisions in line with equity norms, studies that restricted Dictator Games by making them either equitable or inequitable have shown that individuals also often make inequity decisions, for example when they worry about costs for the self or do not want to receive less than others (Güroğlu, Will et al., 2014). Giving behavior and the underlying neural mechanisms also depended on whether such inequity was advantageous or disadvantageous to the self, suggesting that the relative costliness of giving distributions impact giving behavior (Güroğlu, Will et al., 2014). A second important factor relates to the target of giving. Adolescents have a fundamental social motivation for their friends compared to less familiar targets. This is evidenced by a recent study where adults accommodated friend outcomes mainly when this friend was present and could monitor decisions, whereas adolescents did so regardless of whether their friend was present (Powers et al., 2018). This motivation is also apparent in developmental studies assessing giving behavior: with increasing age, adolescents give more to friends, but less to unfamiliar others (Güroğlu, van den Bos et al., 2014). The extent to which adolescents differentiate between giving to friends and unfamiliar others depends on their level of perspective-taking (van de Groep et al., 2020a), a process that develops during adolescence (Dumontheil et al., 2010). A third factor that influences decision-making is whether decisions are made in the presence of peers. It was previously observed that adolescents contribute more to common goods when this behavior is observed or liked by peers (van Hoorn, Dijk et al., 2016), but this study did not examine developmental differences. Developmental studies revealed that adolescents are more susceptible to peer influence effects in a risk-taking task than adults (Smith et al., 2018), although the mere presence of other adolescents does not always result in increased risk-taking (Somerville et al., 2019), suggesting that peer presence effects depend on the specific context (van Hoorn, Fuligni et al., 2016).

The influence of social contextual factors provides a novel perspective on giving in adolescence, but the mechanisms that drive potential developmental changes in giving magnitude, target, and audience effects are not yet well understood. Functional neuroimaging studies can address this by providing insight into the neural correlates of giving. A meta-analysis on strategic and altruistic giving in adults revealed various brain regions that are involved in giving to unfamiliar others, including the medial prefrontal cortex (mPFC), anterior insula (AI), and dorsolateral prefrontal cortex (dlPFC; Cutler and Campbell-Meiklejohn, 2019). Developmental comparison studies demonstrated that adolescents showed stronger activation in the dlPFC and temporal parietal junction (TPJ) when rejecting unfair proposals by unfamiliar others in an Ultimatum Game (Güroğlu et al., 2011). Similar results were observed in a Trust Game, where a first player trusts a division of goods to a second player who can reciprocate. Reciprocating after receiving trust by an unfamiliar other was associated with stronger activation in dlPFC and TPJ with increasing age (van den Bos et al., 2011). These findings suggest that with increasing age adolescents more strongly recruit brain regions implicated in strategic giving, specifically the dlPFC and TPJ (Güroğlu et al., 2011, van den Bos et al., 2011, Will and Güroğlu, 2016).

Recently, researchers suggested that the neural patterns implicated in giving may reveal differential sensitivities to social contexts (Lieberman et al., 2019). In a previous fMRI study with adult participants, we developed a new paradigm in which participants could give a small or large number of valuable coins to friends or unfamiliar peers (Van de Groep et al., 2020b). Half of the trials were performed in an ‘audience present’ condition and the other half of the trials in an ‘anonymous’ condition. Consistent with the meta-analysis by Cutler and Campbell-Meiklejohn (2019), activation in mPFC and AI was dependent on giving magnitude, with stronger activity for relatively generous decisions (van de Groep et al., 2020b). Such activation has been interpreted to reflect norm violation detection, or feelings of empathy and generosity (Crone and Fuligni, 2020, Cutler and Campbell-Meiklejohn, 2019, Feng et al., 2015, Güroğlu et al., 2014, van de Groep et al., 2020b). Furthermore, giving to friends relative to unfamiliar peers was associated with stronger activation in the anterior intraparietal lobe (IPL)/TPJ, possibly indicating that participants took the mental states of others into account (Schreuders et al., 2018, van de Groep et al., 2020b). Finally, being observed by an audience was associated with increased activity in the posterior IPL/TPJ (van de Groep et al., 2020b), consistent with a recent meta-analysis on audience effects (van Hoorn et al., 2019). Other studies in adults have additionally shown that ventral striatum activation is modulated by peer presence in the context of charitable donations. For example, one study observed especially high ventral striatum activation when participant were relatively generous with an audience present, or less generous in the absence of an audience (Izuma et al., 2010).

Taken together, in adults partly separable and partly overlapping neural regions are involved in processing giving magnitude, giving to friends versus distant others, and in giving in the presence of an audience versus anonymously (Braams et al., 2013, Cutler and Campbell-Meiklejohn, 2019, Filkowski et al., 2016, Luo, 2018, Schreuders et al., 2018, Spaans et al., 2019, van de Groep et al., 2020b). The current study used the aforementioned giving paradigm to test the effect of giving magnitude, target familiarity, and audience effects on giving behavior and associated neural activity in adolescence (van de Groep et al., 2020b).

So far, few studies in adolescents have examined whether neural patterns of giving differ depending on giving magnitude, the target, or the presence of an audience. Regarding giving magnitude, one study in 15–18-year-olds showed increased activation in the dACC, ventral midbrain, anterior insula, and cuneus for costly giving versus non-costly rewards (Telzer et al., 2013). Another study including 8–16-year-olds showed enhanced activation in the inferior temporal gyrus and precuneus for costly relative to non-costly giving (Do et al., 2019). With regard to the target, a prior study demonstrated that in adolescents, giving to friends compared to unfamiliar others was associated with stronger activation in the putamen, a subregion of the ventral striatum; the parietal cortex (overlapping or close to the TPJ), and the precentral gyrus (overlapping or close to dlPFC; Schreuders et al., 2019), but this study did not include age comparisons. Second, in an iterated Trust Game paradigm, trusting a trustworthy versus untrustworthy unfamiliar other was associated with an age-related increase in activity in the precuneus/posterior cingulate, whereas the mPFC showed an age-related increase in responsiveness to interactions with an untrustworthy unfamiliar other (Fett et al., 2014). Third, young adolescents with a history of social exclusion and rejection, compared to highly accepted adolescents, showed stronger activity in dlPFC and TPJ when giving generously in a modified Dictator Game to players who previously excluded them in a Cyberball Game (Will et al., 2016). Finally, a study that examined the effects of peer presence on neural activation in adolescents showed increased activation in the mPFC, TPJ, precuneus en STS (Van Hoorn et al., 2016).

Taken together, adolescents and adults recruit similar brain regions when giving in various social contexts, including the ventral striatum, AI, prefrontal cortex (medial and lateral) and social brain regions such as the IPL/TPJ. However, no prior study examined the age-related differences in neural activation throughout adolescence that is implicated in giving in these social contexts. Therefore, this study had three aims, which all address a contextual consideration of giving decisions. The first aim of this study was to examine the effect of giving magnitude on giving behavior and its neural correlates (small vs. large giving). The second aim was to test whether neural correlates of giving considerations were dependent on the target (i.e., a friend versus unfamiliar peer). The third aim was to examine whether giving behavior and neural activation differed as a function of peer presence (giving with an audience versus anonymously). Finally, an important overarching goal was to examine possible interactions with age for each of these three contextual considerations.

### 1.1 Aim & hypotheses of the current study

The current study, including confirmatory hypotheses (outlined below) was pre-registered at the Open Science Framework: https://osf.io/ynpqr/. To examine adolescents’ audience and anonymous giving to friends and unfamiliar peers and the associated neural correlates, we used the giving paradigm previously validated in adults (van de Groep et al., 2020b), in which adolescents divided either a small or large number of coins (giving magnitude manipulation) between themselves and either a friend or unfamiliar peer (target manipulation) in an audience or anonymous context (peer presence manipulation). This task is a modified version of the Dictator Game, which is an economic game traditionally used to study giving behavior (Kahneman et al., 1986, van de Groep et al., 2020b). The task included two giving conditions, in which participants could give away either a relatively small (i.e., less than half of seven coins; low-costly) or large amount (i.e., more than half of seven coins; high-costly; van de Groep et al., 2020b). This approach ensured the inclusion of individuals who would show no or little giving under unrestricted giving conditions (Do et al., 2019, Telzer et al., 2015). Within these task conditions, participants could decide upon the number of coins to give away, which ensured voluntary choices (Gagné, 2003, Murayama et al., 2013), as autonomous decisions give the best indication of generosity (Harbaugh et al., 2007). As such, the current design allowed us to assess the neural comparison between small versus large size giving, even in individuals who show little variability in giving behavior, as well as the neural signature of generosity, by examining the neural activation associated with relative generosity within the small and large giving conditions.

#### 1.1.1 Confirmatory behavioral hypotheses

**(**1) We expected that adolescents would give more to their friends than to unfamiliar peers (main effect target; Güroğlu, van den Bos et al., 2014; van de Groep et al., 2020a). We expected this effect to be strongest in the ‘small amount’ condition compared to the ‘large amount’ condition, because participants’ personal loss is lowest in the small amount condition (target x giving condition interaction). (2) We expected that older adolescents would give more to their friends and less to unfamiliar peers, compared to younger adolescents (target x age interaction; Güroğlu, van den Bos et al., 2014). (3) We expected that participants would donate more in the audience compared to the anonymous condition (main effect peer presence; Krátký et al., 2016, van de Groep et al., 2020b). We explored possible age differences in this effect. (4) Pre-registered hypotheses regarding sex effects were not examined in this study.

#### 1.1.2 Confirmatory brain activation hypotheses

As detailed in our pre-registration, we used both confirmatory ROI analyses and exploratory whole brain analyses to examine the neural activation related to giving in different contexts. The reason for including the exploratory whole brain analyses was to gain insight into neural patterns associated with the newly developed paradigm aside from the a-priori defined ROIs, which can inform hypotheses for future neuroimaging studies. Here, we focused on six pre-registered ROIs that were created using Neurosynth (http://neurosynth.org/; date February 17, 2020). We used this Neurosynth approach, on which more information can be found in the method section, to assure that our confirmatory ROI analyses were grounded in a synthesis of prior fMRI studies. The six ROIs, the mPFC, TPJ, AI, dACC, LPFC, and nAcc, were selected as earlier studies showed involvement of these regions in prosocial decision making, or because they related to more general constructs of mentalizing, decision making, and cognitive control (see https://osf.io/ynpqr/). Our pre-registered expectations regarding neural activation are detailed below. (5) We expected that contrasting the small to large amount condition would result in activation in at least one of the following brain regions: the mPFC and the AI (van de Groep et al., 2020b). (6) We expected that contrasting the condition in which adolescents give to a friend to the condition in which adolescents give to an unfamiliar peer would result in activation in at least one of the following brain regions: the TPJ and ventral striatum (Schreuders et al., 2019, van de Groep et al., 2020b). (7) We expected that being observed in the audience condition would result in increased activity in brain regions that are part of the social brain network, including the TPJ (Van Hoorn et al., 2016). (8) No hypotheses were pre-registered about age-related differences in neural activation. However, based on prior literature showing increased dlPFC and TPJ activation with increasing age for fair decisions, we expected increased activation with increasing age in these regions for relatively generous decisions (Güroğlu et al., 2011, van den Bos et al., 2011). Furthermore, based on prior literature we expected age-related increases in activation for giving to a friend versus unfamiliar peer in the dlPFC and TPJ (Güroğlu et al., 2011, Schreuders et al., 2019, van de Groep et al., 2020b, van den Bos et al., 2011).

## 2 Method

### 2.1 Sample and participant selection

In total, 142 adolescents participated in this cross-sectional study, which is the first measurement wave of a three-wave longitudinal project on the development of prosocial behavior in adolescence called ‘Brainlinks’. Participants were excluded from further analysis when they did not perform or finish the fMRI task (*N* = 2) or showed head movement (≥ 3 mm) during the fMRI task (*N* = 11). The final sample consisted of 128 adolescents (81 females), between the ages of 9 – 19 (*M*_*age*_ = 14.78, *SD*_*age*_ = 2.65; age range 9.00 – 18.89 years). Participants were recruited through local and online advertisements and provided written informed consent. For minors (i.e., ages 15 and younger), both parents also provided written informed consent. Participants had normal or corrected-to-normal vision, and no diagnosed intellectual disability (IQ < 70). Participants were screened by means of a checklist for neurological or psychiatric disorders and MRI contraindications via a private telephone conversation. The local medical ethical committee approved the study. Participants received €20 (ages < 12 years) or €30 (ages ≥ 12 years) and small presents for their participation, plus additional earnings from the fMRI task and other tasks that were performed as part of the larger study protocol.

### 2.2 Materials: fMRI giving task

#### 2.2.1 Giving magnitude: giving small and large amounts

**Fig. 1:**
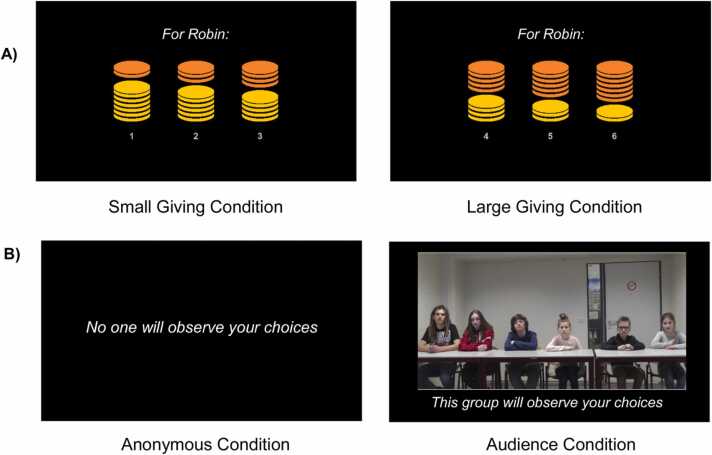
(A) The small and large giving conditions of the task, in which participants could give away 1, 2, or 3 coins, or 4, 5, or 6 coins, respectively (depicted in orange). Participants would keep the remainder of the 7 coins (depicted in yellow) to themselves. The name of the target (which could either be a friend or unfamiliar peer) was displayed at the top of the screen for each trial. (B) In two out of four blocks of the giving task, participants made anonymous giving choices. In the other two blocks, participants were aware that the peer audience depicted on the screen would observe their choices later in time (i.e., the audience condition). Note that the screens indicating whether blocks were audience or anonymous were only displayed at the start of each block, not during trials. Audience and anonymous blocks were presented in counterbalanced order.

To assess the neural correlates of giving behavior, we used a modified fMRI version of the Dictator Game previously validated in adults (Kahneman et al., 1986, van de Groep et al., 2020b). Participants divided 7 coins between themselves and another person - who could not reject the decision - in either a small or large giving condition. Giving was operationalized as the number of given coins. In the small giving condition, participants could give away 1, 2, or 3 out of 7 coins. In the large giving condition, participants could give away 4, 5, or 6 out of 7 coins. Participants could not give 0 coins, 7 coins, or make an equal split to warrant comparability between the small and large giving condition (see Fig. 1).

#### 2.2.2 Familiarity of the target: friend and unfamiliar peer

The target of giving was either the participant’s closest friend (same-sex, similar-age) or an unfamiliar peer (same-sex, similar-age, anonymized participant of the same study). For each trial, the name of the target was displayed at the top of the screen (see Fig. 1). Participants were instructed that the coins they divided in each trial were worth actual money (i.e., 20 eurocents each) and that the computer would randomly select a few trials of the task to determine the payout of the participant, friend, and unfamiliar other. Accordingly, participants received a payment for themselves (*M* = €.80, *SD* = €.12) and their friend (*M* = €.68, *SD* = €.08), and experimenters transferred the payment to the unfamiliar other (i.e., another participant of the current study; *M* = €.61, *SD* = €.09).

#### 2.2.3 Peer presence: anonymous and audience giving

To assess the effects of peer presence on giving, the task consisted of two blocks in which participants made anonymous choices, and two blocks in which participants’ choices were evaluated by peers later in time (see Fig. 1). The order of anonymous and audience blocks was counterbalanced across participants. In a practice session prior to the MRI session, participants viewed a video clip of six peers (three males and three females, aged 9–19) with neutral expressions. To the awareness of participants, these six peers were invited after study completion to observe and evaluate choices of participants. Trials in the anonymous blocks were not shown to anyone, as – beknown to participants - experimenters covered the screen in the control room.

#### 2.2.4 Task duration and stimuli

The task was presented in the MRI scanner via E-prime version 2 (Schneider et al., 2012). Each block began with a screen showing, for 4000-milliseconds, whether it was an anonymous or audience block. These screens were displayed at the start of each block, and not during trials, to avoid differences in visual complexity and thus neural activation between audience and anonymous trials. Within a block, each trial started with a jittered fixation cross for 0 – 4400 ms (*M* = 550 ms) which was optimized using OptSeq (Dale, 1999). Hereafter, three possible giving options were shown (i.e., giving 1, 2, or 3 coins in the small giving condition, and 4, 5, or 6 coins in the large giving condition). Participants had to choose within 2000 ms how many coins to give by pressing a response button with their right index-, middle-, or ring finger. Choices were confirmed for 1000 ms via a white frame around the selected choice. In case participants did not press in time, a ‘too late’ screen was shown for 1000 ms and these trials were excluded from analysis.

#### 2.2.5 Order of blocks and trials

The task consisted of four blocks with 40 trials per block, resulting in 160 trials in total. All combinations of conditions (giving magnitude, target, and peer presence) were equally distributed across trials. The order of trials was optimized using OptSeq (Dale, 1999). There was a short break between the second and third block of the task (i.e., in between runs). The total length of the task, excluding instructions and pauses, was approximately 15 min. Before scanning, participants performed a practice version of the task consisting of four trials per condition in randomized order.

#### 2.2.6 Manipulation check

To check whether participants differentiated between targets, they rated how important the friend and unfamiliar peer were to them and how much they liked them on a 7-point scale, ranging from 1 (not at all) to 7 (very much).

#### 2.2.7 Giving behavior

We measured participants’ giving behavior with a button response box, where pressing the left button was coded as 1, the middle as 2, and the right as 3. To enable a comparison of relative generosity in the small and large giving conditions, scores were transformed to percentages. As such, the lowest number of coins given (1 and 4) were recoded as 33.33%, the middle number of coins given (2 and 5) as 66.66%, and the highest number of coins given (3 and 6) as 100%. As detailed in our pre-registration, we used these scores to examine average giving behavior and whether giving varied as a function of task conditions. We also examined whether there were reaction time differences between conditions. Furthermore, we examined age effects (i.e., linear and quadratic) on giving.

Note that a Bonferroni correction was applied to the behavioral analyses to account for multiple testing, resulting in a corrected alpha of .01 (*p* = .05 divided by 5 behavioral analyses is *p* = .01). While most results were significant after this correction, some were not. For these analyses, we report that they are significant at an uncorrected threshold.

### 2.3 Procedure

After expressing interest in the study, participants received information by telephone and a digital information letter. After agreeing to take part, participants filled out two questionnaires prior to their laboratory visit, which included self-report measures on prosocial behaviors and personality. During their visit, participants first received instructions about the visit, MRI scanner, and measurements. The MRI session consisted of a functional scan, structural scan, two fMRI tasks, and a diffusion tensor imaging scan. Finally, participants filled out questionnaires and performed tasks outside the MRI scanner.

### 2.4 MRI data acquisition

We acquired MRI scans using a 3T MRI scanner (Philips Achieva TX, Erlangen, Germany) with a standard whole-head coil. Stimuli were shown using a screen, which participants could see through a mirror attached to the head coil. Functional scans were acquired during two runs, which consisted of 178 and 205 dynamic scans, respectively. We collected T2* weighted gradient echo planar images (EPI) (TR = 2.2 s, TE = 30 ms, flip angle 8°, sequential acquisition: 38 slices, voxel size = 2.75 ×2.75 ×2.75 mm, 80 ×80 matrix, field of view [FOV] = 220 × 220 × 115 mm). Before the start of each functional run, 5 dummy scans were acquired. For anatomical reference, a high-resolution 3D T1-weighted anatomical image was collected prior to the functional scans (TR = 7.9 ms, TE = 3.5 ms, flip angle 8°, 3D matrix size for 3D acquisitions: 228 ×177 x 155 slices, axial slice orientation, voxel size: 1.1 ×1.1 ×1.1 mm, FOV = 250 ×196 x 170 mm). T1 stabilization dummy scans were automatically discarded by the scanner. This scan’s length was 4 min and 12 s. All scans were acquired using a fast field echo pulse sequence. To avoid head motion, participants’ head motion was limited with foam inserts at both sides of the head when possible. Before exclusion of participants who showed excessive head motion, movement was as follows: movement range: .00 – 11.30 mm, *M* = 0.10, *SD* = 0.12. Movement for the final sample was as follows: .00 – 2.97 mm, *M* = 0.08, *SD* = 0.08.

### 2.5 MRI data analysis

#### 2.5.1 Preprocessing

MRI data analysis was performed using SPM8 (Welcome Department of Cognitive Neurology, London, United Kingdom). Functional images were preprocessed using the following steps: realignment, slice-time correction, spatial normalization using segmentation parameters, and spatial smoothing with a 6-mm FWHM isotropic Gaussian kernel. The normalization algorithm used a 12-parameter affine transform with a nonlinear transformation involving cosine basis functions and resampled the volumes to 3-mm cubic voxels. Templates were based on MNI-305 stereotaxic space. All functional scans were corrected for excessive head motion using 6 parameters.

#### 2.5.2 General linear model

To perform first-level individual analyses, we used the general linear model in SPM8. The fMRI time series were modeled as a series of zero duration events time-locked to stimulus onset and convolved with the hemodynamic response function (HRF). Note that a reaction times model showed highly similar results. We used the modelled events (i.e., giving magnitude condition: small vs. large; target: friend vs. unfamiliar peer; peer presence: audience vs anonymous giving) as regressors in a general linear model, alongside a basic set of cosine functions that high-pass filtered the data (cutoff: 120 s). The start screens, in which the type of block (anonymous or audience present) was indicated, were modelled separately. In addition, we included six motion parameters as nuisance regressors. Trials on which participants did not respond were modelled separately as covariate of no interest and were excluded from analyses. We used the least-square parameter estimates of the height of the best-fitting canonical hemodynamic response function for each condition in pair-wise contrasts. These pairwise comparisons led to subject-specific contrast images, which we submitted to second-level group analyses. We followed all analyses steps as detailed in our pre-registration on the Open Science Framework https://osf.io/ynpqr/, with three exceptions. First, we did not include B0 field maps, as inclusion was not compatible with our MRI analysis pipeline. Second, we did not use the scrubbing option. Instead of scrubbing, we excluded participants who moved more than 3 mm during the functional scans. Although additional movement control procedures, such as scrubbing (i.e., accounting for volumes in which movement exceeds a certain threshold, as described by Siegel et al., 2014) are important to consider even after excluding participants with > 3 mm movement, such decisions should be weighed against power loss by removing data. Therefore, this study strictly excluded participants based on movement (>3 mm), and added motion parameters to the design, but did not use additional scrubbing as power in each cell would be significantly reduced by data loss. Full information on movement parameters in the current sample is presented in Supplement 1. Third, we extracted the NAcc ROIs from the Harvard-Oxford subcortical atlas instead of the AAL atlas as described in the pre-registration as the AAL atlas only includes ROIs of the whole caudate and not the NAcc specifically.

#### 2.5.3 Confirmatory ROI analyses

To test for neural differences related to giving condition and target, we created 6 ROIs using the MarsBaR toolbox (Brett et al., 2002; http://MarsBaR.sourceforge.net/) for SPM8 for which we extracted parameter estimates. We defined our ROIs (i.e., the mPFC, TPJ, AI, dACC, LPFC, and nAcc) using Neurosynth (http://neurosynth.org/; date February 17, 2020). The center of mass for each of the ROIs was extracted from Neurosynth meta-analyses using target words: “mentalizing” for the mPFC and bilateral TPJ, “decision making” for the AI and dorsal anterior cingulate cortex (dACC), and “cognitive control” for the LPFC) Consecutively, we used MarsBaR to build 10 mm spheres around the center of mass extracted for each ROI. NAcc ROIs were extracted from the Harvard-Oxford subcortical atlas with a threshold of 40%. The left nAcc consisted of 28 voxels [ x = −9.57, y = 11.70, z = −7.10] and the right nAcc consisted of 26 voxels [ x = 9.45, y = 12.60, z = −6.69]. An image with all ROIs was uploaded to NeuroVault (Gorgolewski et al., 2015), see ‘Other’ maps on https://neurovault.org/collections/ICTLVEUU/. For each of the ROIs, we tested for main effects and interactions of task-conditions, using 2x2x2 (i.e., giving condition x target x peer presence) repeated measures ANOVAs. Given the focus on six ROIs, a Bonferroni corrected alpha of .008 was used to account for multiple testing. That is, we used a *p* value of.05 divided by the number of ROIs: .05/6 = 0.008. To examine age effects, linear age and quadratic age were added to the repeated measures ANOVAs in a stepwise manner.

#### 2.5.4 Whole brain analysis examining task conditions

We performed a 2 (giving condition: small or large) x 2 (target: friend or unfamiliar peer) x2 (peer presence: audience vs anonymous giving) ANOVA to explore neural responses across the whole brain at the group level. We examined the following whole-brain contrasts: ‘small giving condition versus large giving condition’, ‘friend versus unfamiliar peer’, and ‘audience versus anonymous giving’, as well as the reverse contrasts, and tested for possible interaction effects between conditions. Task-related responses were considered significant when they surpassed false discovery rate (FDR) cluster correction of *p* < .05, with an initial uncorrected threshold of *p* < .001 (Woo et al., 2014). All reported whole brain analyses are available on NeuroVault, see group maps on https://neurovault.org/collections/ICTLVEUU/.

#### 2.5.5 Exploratory age-related whole brain analysis

To explore age effects that were not related to the predefined ROIs, linear age and quadratic age were added to the whole-brain contrasts.

#### 2.5.6 Exploratory generosity-related whole brain analysis

Our prior study in young adults that used the same experimental paradigm showed that relatively generous response options within the small and large giving condition (i.e., giving 2 or 3 coins, or 5 or 6 coins, as compared to 1 or 4 coins, respectively) resulted in increased activation in the mPFC and right AI. Therefore, although not specified in our pre-registration, we also examined minimal vs. generous giving and the reverse contrasts within the small (i.e., giving 1 vs 2/3 coins) and large (i.e., giving 4 vs. 5/6 coins) giving conditions. This analysis could only be performed for participants who showed every response option within the giving condition, resulting in 107 participants for the small giving condition and 86 participants for the large giving condition. To maximize the number of trials in this analysis, we collapsed across target and peer presence conditions. To examine age effects, linear age and quadratic age were added to the whole-brain contrasts.

## 3 Results

We performed assumption checks for all analyses. For all behavioral variables and some neural variables, Kolmogorov-Smirnov and Shapiro-Wilk tests of normality were significant, indicating deviations from normality (i.e., absolute *z*-values > 3.29 and *p*’s ≤ 0.05). However, as skewness values were all lower than 2, and square, square root, cube root, inverse, and log transformations did not improve normality, we report untransformed values for all analyses. No other violations of assumptions were observed. There were a few outliers as assessed by inspection of a boxplot (i.e., values greater than 3 box-lengths from the edge of the box), which were therefore winsorized (Tabachnik and Fidell, 2013). Specifically, three participants were outliers on one variable each. We report the winsorized results, but the results were similar with and without winsorizing. For each of the repeated measures ANOVAs reported below, we tested all main and interaction effects.

### 3.1 Behavioral results

#### 3.1.1 Manipulation checks

Participants rated their friend as more important (*M* = 6.46, *SD* =0.83) than the unfamiliar peer (*M* = 3.37, *SD* = 1.47), as indicated by a paired-samples t-test, *t*(127) = 22.97, *p* < .001. Participants also liked their friend (*M =* 6.63, *SD* =0.60) more than the unfamiliar peer (*M* = 4.06, *SD* =0.67), *t*(127) = 32.11, *p* < .001. Therefore, the manipulation checks confirmed that participants differentiated between friends and unfamiliar peers regarding importance and liking.

#### 3.1.2 Giving behavior

**Fig. 2:**
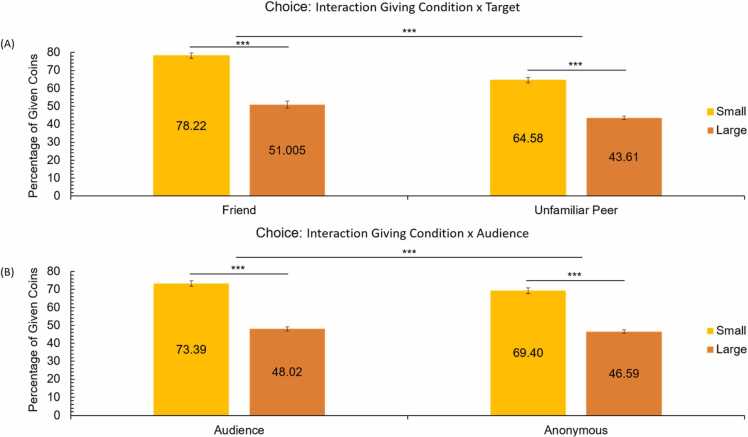
(A) Mean percentage of coins given to a friend and unfamiliar peer in the small and large giving conditions. (B) Mean percentage of coins given in the small and large giving condition, for each of the peer presence (audience vs. anonymous) conditions.

To examine the effects of task conditions on giving behavior, we performed a repeated measures ANOVA with giving condition (small vs. large), target (friend vs. unfamiliar peer), and peer presence (audience vs. anonymous giving) as within-subject variables and giving (in percentages) as dependent variable. There was a main effect of giving condition, *F*(1, 127) = 153.23, *p* < .001, *η*²_p_ = .55, such that participants gave relatively more in the small (*M* = 71.39, *SD* = 17.47) compared to the large giving condition (*M* = 47.31, *SD* = 11.82). There was also a main effect of target, *F*(1, 127) = 99.97, *p* < .001, *η*²_p_ = .44, and a two-way interaction between giving condition and target, *F*(1, 127) = 21.41, *p* < .001, *η*²_p_ = .14. As shown in Fig. 2, participants gave more to a friend (*M* = 64.61, *SD* = 11.24) than an unfamiliar peer (*M* = 54.09, *SD* = 12.13), but this difference was more pronounced in the small giving condition (*M*_*friend*_ = 78.21, *SD*_*friend*_ *=* 16.67*; M*_*unfamiliar peer*_ = 64.57*, SD*_*unfamiliar* peer_ = 21.46) compared to the large giving condition (*M*_*friend*_ =51.00, *SD*_*friend*_ *=* 15.50*; M*_*unfamiliar peer*_ = 43.61*, SD*_*unfamiliar* peer_ = 10.55).

Finally, there was a main effect of peer presence, *F*(1, 127) = 43.11, *p* < .001, *η*²_p_ = .25, which was qualified by a two-way interaction between giving condition and peer presence, *F*(1, 127) = 12.36, *p* = .001, *η*²_p_ = .09. As can be seen in Fig. 2, participants gave more in the audience condition than in the anonymous condition, but the audience effect was more pronounced in the small giving condition (*M*_*audience present*_ = 73.39, *SD*_*a*__*udience present*_ *=* 17.35*; M*_*audience absent*_ = 69.40*, SD*_*audience* absent_ = 18.21) compared to the large giving condition (*M*_*audience present*_ = 48.02, *SD*_*audience present*_ *=* 13.03*; M*_*audience absent*_ = 46.60*, SD*_*audience* absent_ = 11.25). There was no interaction between target and audience, nor was there a three-way interaction.

#### 3.1.3 Age effects on giving

**Fig. 3:**
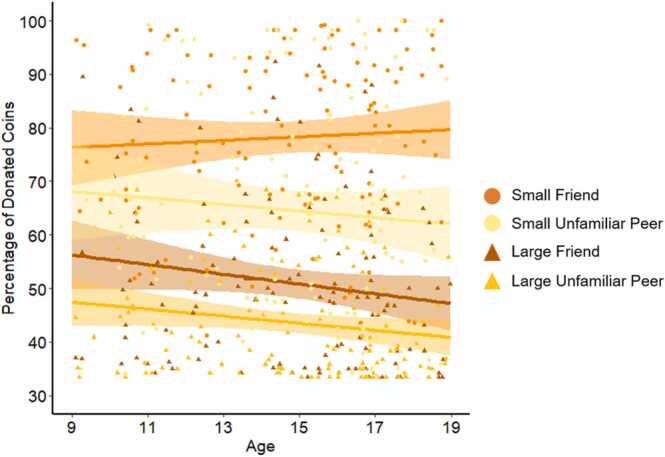
Associations between age and the mean percentage of given coins for friend and unfamiliar peer in the small and large giving condition.

To examine effects of age on giving behavior, linear and quadratic age were added as covariates to the repeated-measures ANOVA. This analysis yielded a three-way interaction between age, giving condition, and target, *F*(1, 126) = 5.66, *p* = .019, *η*²_p_ = .04 (uncorrected for multiple comparisons). Follow-up analyses showed a trend towards increased differentiation between targets in the small giving condition (Δ*B*_*friend-unfamiliar peer*_ *=*.94; *F*(1, 126) = 3.17, *p* = .077, *η*²_p_ = .03) compared to the large giving condition (Δ*B*_*friend-unfamiliar peer*_ *=*.18; *F*(1, 126) = 0.38, *p* = .537, *η*²_p_ = .00); see Fig. 3. In the large giving condition, there was a general age-related decrease in giving behavior (*r* = −0.18, *p* = .047). There were no other linear age effects, nor where there any quadratic age effects.

#### 3.1.4 Reaction times

**Fig. 4:**
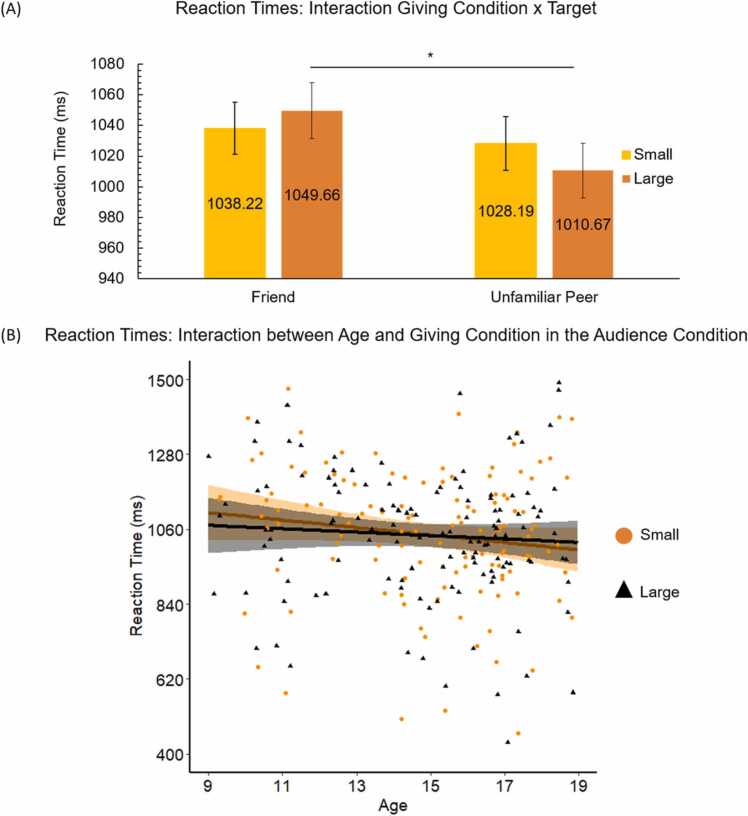
(A) Reaction times in milliseconds associated with giving to a friend and unfamiliar peer in the small and large giving conditions. Error bars represent the standard error. (B) Associations between age and reaction times in milliseconds in the small and large giving conditions with the audience present. There was a larger age-related decrease in RT for the small compared to the large condition when audience was present.

To examine differences in reaction times depending on task conditions, we performed a repeated measures ANOVA with giving condition (small vs. large), target (friend vs. unfamiliar peer), and peer presence (audience vs. anonymous giving) as within-subject variables and reaction time in milliseconds as dependent variable. We found a main effect of target, *F*(1, 127) = 15.86, *p* < .001, *η*²_p_ = .11, which was qualified by a giving condition x target interaction, *F*(1, 127) = 8.37, *p* = .004, *η*²_p_ = .06. Follow-up analyses revealed no differences in RTs between the friend and unfamiliar peer in the small giving condition, *F*(1, 127) = 1.55, *p* = .215, *η*²_p_ = .00, *M*_*friend*_ = 1038.22, *SD* = 176.58, *M*_*unfamiliar peer*_ = 1028.19, *SD* = 192.94, but longer RTs for giving to a friend compared to an unfamiliar peer in the large giving condition, *F*(1, 127) = 5.60, *p* = .020, *η*²_p_ = .04, *M*_*friend*_ = 1049.66, *SD* = 186.30, *M*_*unfamiliar peer*_ = 1028.19, *SD* = 192.94 (see Fig. 4).

With regard to peer presence, results were only significant at an uncorrected threshold (i.e., *p* = .05): there was a main effect of peer presence, *F*(1, 127) = 5.33, *p* = .023, *η*²_p_ = .04, such that reaction times were longer in the audience (*M* = 1048.34, *SD* = 195.03) compared to the anonymous (*M* = 1023.79, *SD* = 184.62) condition. Adding linear age to the analysis revealed a three-way interaction between audience, giving condition, and age, *F*(1, 126) = 6.02, *p* = .016, *η*²_p_ = .05. Follow up analyses revealed an interaction between giving condition and age in the audience, *F*(1, 126) = 4.27, *p* = .041, *η*²_p_ = .03 but not the anonymous condition¸ *p* = .465. As shown in Fig. 4, there was a larger age-related decrease in RT for the small compared to the large condition when audience was present. There were no other linear age effects, and no quadratic age effects.

### 3.2 Neural results

#### 3.2.1 Confirmatory ROI analyses

**Fig. 5:**
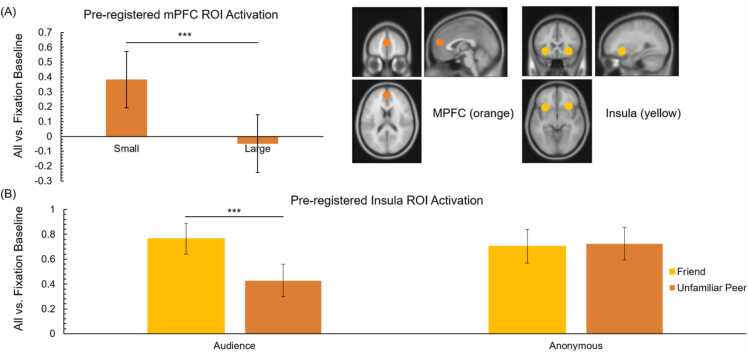
Task condition effects in pre-defined ROIs. (A) The mPFC showed more activation (i.e., less de-activation) in the small compared to large giving condition. (B) The insula showed an interaction between target and audience, such that there was a difference between activation for the friend and unfamiliar peer in the audience but not anonymous condition. Error bars represent the standard error. Abbreviations: mPFC = medial prefrontal cortex.

For the confirmatory ROI analyses, we performed similar repeated measures ANOVAs with giving condition (small vs. large), target (friend vs. unfamiliar peer), and peer presence (audience vs. anonymous giving) as within-subject variables and ROI activity as dependent variable. Results are outlined below for each ROI. For the mPFC, there was a main effect of giving condition, *F* (1, 127) = 12.18, *p* < .001, *η*²_p_ = .09, such that activation was higher in the small (*M* =0.38, *SD* = 2.14) compared to large giving condition *(M* = −0.05, *SD* = 2.20), see Fig. 5A. There were no significant effects of target and peer presence.

For the AI, there was a main effect of target, *F* (1, 127) = 7.64, *p* = .007, *η*²_p_ = .06, such that activity was higher for the friend than unfamiliar peer. This effect was qualified by an interaction between target and audience, *F* (1, 127) = 10.37, *p* = .002, *η*²_p_ = .08. As can be seen in Fig. 5B, there was a difference in AI activation between the friend and unfamiliar peer in the audience giving condition, *F* (1, 127) = 13.91, *p* < .001, *η*²_p_ = .01, *M*_*friend*_ = 0.77, *SD*_*friend*_ *=* 1.40*; M*_*unfamilar_peer*_= 0.43*, SD*_*unfamiliar_peer*_ = 1.53, but not in the anonymous condition, *M*_*friend*_ = 0.71, *SD*_*friend*_ *=* 1.48*; M*_*unfamilar_peer*_= 0.72*, SD*_*unfamiliar_peer*_ = 1.46. For the AI ROI, there was no effect of giving condition and there were no further interactions.

Finally, contrary to predictions, there were no significant effects for the nAcc, dACC TPJ and LPFC. See Supplement 2 for an overview of activation in all ROIs for each of the task conditions.

#### 3.2.2 Confirmatory ROI age analyses

Adding linear and quadratic age to the confirmatory ROI analyses revealed no age effects.

#### 3.2.3 Whole brain analysis examining task conditions

To examine neural responses at the whole brain level, we performed a whole-brain full factorial ANOVA with giving condition (small vs. large), target (friend vs. unfamiliar peer) and peer presence (audience vs. anonymous giving) as within-subject factors.

**Fig. 6:**
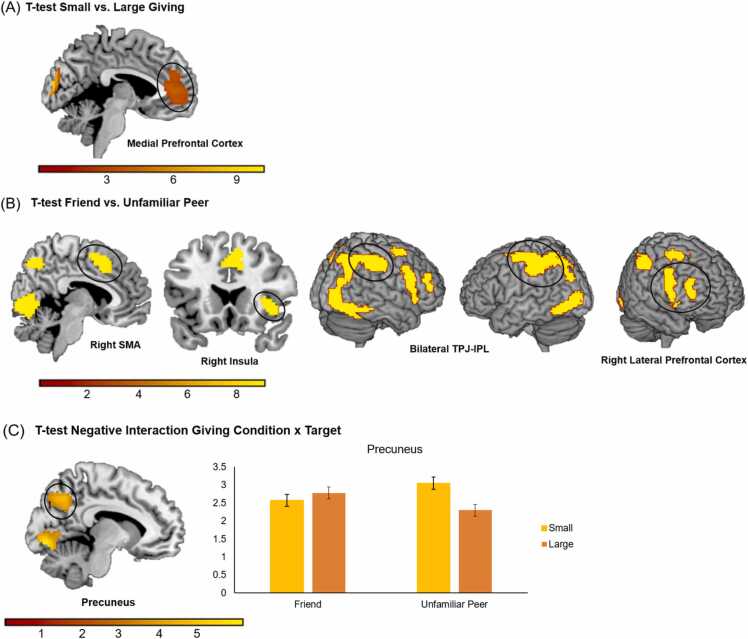
(A) Whole-brain t-test for the small versus large giving condition. (B) Whole-brain t-test for friend versus unfamiliar peer. Results are displayed with a primary voxel-wise threshold of p < .001 (uncorrected) and FDR cluster correction of p < .05. (C) Activation in the precuneus in the small and large giving condition for friend and unfamiliar peer, showing an interaction between giving condition and target. Specifically, activation was higher for the friend in the large giving condition, and higher for the unfamiliar peer in the small giving condition. Error bars represent the standard error.

The overall *F*-test of giving condition revealed activation in the mPFC, left postcentral gyrus, right precentral gyrus, left lingual gyrus, right precuneus and several other regions. Paired samples t-tests showed that the mPFC (see Fig. 6A) and left calcarine were more active in the small compared to large giving condition, *t’s* ≥ 4.85*, p*'s ≤ 0.001. The left lingual, right precentral gyrus, left postcentral gyrus, and right precuneus were more active in the large compared to small giving condition, *t’s* ≥ 4.34*, p*'s ≤ 0.001, see Table 1.

**Table 1:** MNI coordinates of local maxima activated for the F-test and t-test for small > large giving condition. Results were calculated using a primary voxel-wise threshold of p < .001 (uncorrected), with a cluster corrected threshold of *p* < .05 FDR corrected.

Area of activation	MNI Coordinates			Test statistic	Cluster Size
	x	y	z	*F/t*	
*F-test Giving Condition (FDRc < 0.001 = 21.57)*					
Left Lingual	-9	-79	-5	332.90	1652
Right Anterior Cingulum (i.e., mPFC)	6	44	4	23.51	423
Right Precentral	54	-13	46	21.57	159
Left Postcentral	-48	-19	49	43.22	108
Right Frontal Superior Medial	12	-58	58	18.84	51
Right Inferior Occipital	30	-91	-5	41.50	42
Left Rolandic Operculum	-45	-13	16	16.00	37
Right Frontal Inferior Orbital	36	23	-14	15.28	34
Right Temporal Middle	66	-31	-2	17.72	30
Right Precuneus	12	56	22	18.11	22
*t-test Small > Large Giving Condition (FDRc < 0.001 = 553)*					
Left Calcarine	-6	-94	10	10.19	735
Right Anterior Cingulum (i.e., mPFC)	6	44	4	4.85	553
*t-test Large > Small Giving Condition (FDRc < 0.001 = 95)*					
Left Lingual	-9	-79	-5	18.25	1005
Right Precentral	54	-13	46	4.64	204
Left Postcentral	-48	-19	49	6.57	122
Right Precuneus	12	-58	58	4.34	95

*Note:* Names were based on the aal toolbox in SPM. For functional regions discussed throughout the paper, both the aal label and functional label (between brackets) are displayed. See https://neurovault.org/collections/ICTLVEUU/ for a full, unthresholded overview of activation.

The main *F*-test of target revealed activation in the bilateral TPJ, right SMA (i.e., supplementary motor area), right lateral PFC, left precuneus, left caudate, right insula and various other regions, see Table 2. Paired samples t-tests revealed that all brain regions were more active for friend than unfamiliar peer, *t*'s ≥ 3.91, *p*'s ≤ 0.001, with bilateral TPJ, right SMA, and right lateral PFC surviving FDR cluster correction, see Fig. 6B.

**Table 2:** MNI coordinates of local maxima activated for the F-test and t-test for friend > unfamiliar peer. Results were calculated using a primary voxel-wise threshold of p < .001 (uncorrected), with a cluster corrected threshold of *p* < .05 FDR corrected. The reversed unfamiliar peer > friend contrast did not result in significant effects.

Area of activation	MNI Coordinates			Test statistic	Cluster Size
	x	y	z	*F/t*	
*F-test Target (FDRc < 0.001 = 20.46)*					
Left Precentral (i.e., left TPJ within this cluster)	-36	-28	58	37.35	974
Right Lingual	12	-79	-8	90.63	840
Right SupraMarginal (i.e., right TPJ within this cluster)	45	-37	43	34.76	765
Right Supplementary Motor Area	9	8	52	21.44	254
Left Occipital Middle	-30	-91	1	34.04	219
Right Frontal Inferior Operculum (i.e., right LPFC)	51	8	28	21.47	173
Right Frontal Middle	30	-1	58	19.31	59
Right Frontal Middle	42	32	22	15.31	59
Right Insula	39	20	-2	20.94	56
Left Caudate	-21	26	1	27.12	48
Right Thalamus	24	-28	-2	22.45	29
Left Lingual	-9	-76	-8	17.85	27
Left Precentral	-42	-1	34	17.11	25
Left Cingulum Anterior	-6	29	16	14.83	25
*t-test Friend > Unfamiliar Peer (FDRc < 0.001 = 89)*					
Right Lingual (left and right TPJ within this cluster)	12	-79	-8	9.52	2989
Left Precentral (i.e., left TPJ within this cluster)*	-36	-28	58	6.11	
Right SupraMarginal (i.e., right TPJ within this cluster)*	45	-37	43	5.90	
Right Supplementary Motor Area	9	8	52	4.63	324
Right Frontal Inferior Operculum (i.e., right LPFC)	51	8	28	4.63	305
Right Insula* *	39	20	-2	4.58	
Left Occipital Middle	-30	-91	1	5.83	256
Right Frontal Middle	30	-1	58	4.39	94
Right Frontal Middle	42	32	22	3.91	89

*Note:* Names were based on the aal toolbox in SPM. For functional regions discussed throughout the paper, both the aal label and functional label (between brackets) are displayed. See https://neurovault.org/collections/ICTLVEUU/ for a full, unthresholded overview of activation. * These local maxima are part of the larger cluster of 2989 voxels. * * The local maximum for the insula region is part of the larger LPFC cluster of 305 voxels.

The main effect (i.e., *F*-test) of audience revealed activation in the left lingual and right fusiform gyrus, see Table 3. Paired samples t-tests revealed that all brain regions were more active for the audience compared to anonymous condition, *t*'s ≥ 3.81, *p*'s ≤ 0.001.

**Table 3:** MNI coordinates of local maxima activated for the F-test and t-test for audience > anonymous giving. Results were calculated using a primary voxel-wise threshold of p < .001 (uncorrected), with a cluster corrected threshold of *p* < .05 FDR corrected. The reversed anonymous > audience contrast did not result in significant effects.

Area of activation	MNI Coordinates			Test statistic	Cluster Size
	x	y	z	*F/t*	
*F-test Audience (FDRc < 0.001 = 20.47)*					
Left Lingual	-24	-52	-8	20.47	96
Right Fusiform	27	-40	-14	14.55	38
*t-test Audience > Anonymous (FDRc < 0.001 = 77)*					
Left Lingual	-24	-52	-8	4.52	137
Right Fusiform	27	-40	-14	3.81	77

*Note:* Names were based on the aal toolbox in SPM See https://neurovault.org/collections/ICTLVEUU/ for a full, unthresholded overview of activation.

As indicated by an F-test, there was an interaction between giving condition and target, see Table 4. Follow up *t*-tests revealed an interaction in the precuneus, left lingual gyrus, and right fusiform gyrus. To examine the effect in the precuneus in more detail, we used the Marsbar toolbox to extract activation for this cluster. As can be seen in Fig. 6C, in the precuneus, activation in the small giving condition was higher for unfamiliar peer (*M* = 3.05, SD = 1.98) compared to friend (*M* = 2.58, SD = 1.91), *F*(1, 127) = 12.54, *p* = .001, *η*²_p_ = .09; whereas in the large giving condition activation was higher for friend (*M* = 2.78, SD = 1.84) than unfamiliar peer (*M* = 2.30, SD =0.1.82), *F*(1, 127) = 11.69, *p* = .001, *η*²_p_ = .08.

**Table 4:** MNI coordinates of local maxima activated for the F-test and (negative) t-test for the interaction between giving condition and target. Results were calculated using a primary voxel-wise threshold of p < .001 (uncorrected), with a cluster corrected threshold of *p* < .05 FDR corrected. The reversed positive contrast did not result in significant effects.

Area of activation	MNI Coordinates			Test statistic	Cluster Size
	x	y	z	*F/t*	
*F-test Interaction Giving Condition x Target (FDRc < 0.001 = 26.54)*					
Left Precuneus	-18	-58	34	26.54	874
Left Lingual	-12	-82	-8	35.83	413
Right Fusiform	33	-67	1	22.84	198
Right Insula	36	-16	28	18.18	35
*t-test Negative Interaction Giving Condition x Target (FDRc < 0.001 = 298)*					
Left Precuneus	-18	-58	34	5.15	1234
Left Lingual	-12	-82	-8	5.99	539
Right Fusiform	33	-67	1	4.78	298

*Note:* Names were based on the aal toolbox in SPM. See https://neurovault.org/collections/ICTLVEUU/ for a full, unthresholded overview of activation.

#### 3.2.4 Exploratory Whole Brain Age Analyses

**Fig. 7:**
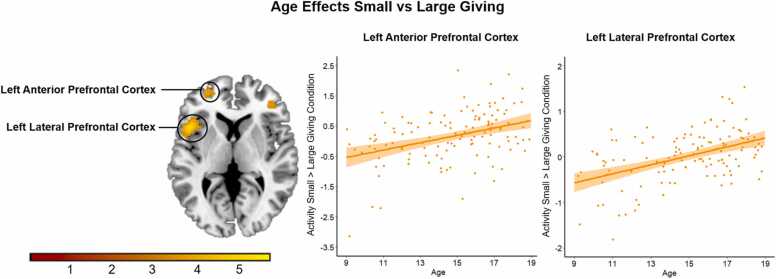
Positive linear age association with activation in the left lateral and anterior prefrontal cortex for the contrast ‘small versus large giving’.

Regarding magnitude giving condition, there was a positive linear association between age and activation in lateral and anterior PFC regions, see Table 5. To investigate the effect in the left anterior and left lateral PFC in more detail we used the Marsbar toolbox to extract activation for these clusters. As shown in Fig. 7, older adolescents recruited the left lateral and anterior PFC more strongly when giving small versus large amounts. There were no significant age effects relating to the whole brain contrasts for target or peer presence.

**Table 5:** MNI coordinates of local maxima activated for linear age regressions for giving condition and target. Results were calculated using a primary voxel-wise threshold of p < .001 (uncorrected), with a cluster corrected threshold of *p* < .05 FDR corrected.

Area of activation	MNI Coordinates			Test statistic	Cluster Size
	x	y	z	*t*	
*Positive Regression Linear Age: Giving Condition (FDRc < 0.001 = 110)*					
Left Frontal Inferior Operculum (i.e., left LPFC)	-48	14	7	5.69	365
Left Frontal Middle (i.e., anterior PFC)	-33	47	13	4.09	114
Right Frontal Middle	39	41	1	4.10	110
*Positive Regression Linear Age: Target (FDRc < 0.001 = 71)*					
Left Thalamus	-6	-19	-8	4.31	71

*Note:* Names were based on the aal toolbox in SPM. For functional regions discussed throughout the paper, both the aal label and functional label (between brackets) are displayed. See https://neurovault.org/collections/ICTLVEUU/ for a full, unthresholded overview of activation.

#### 3.2.5 Exploratory whole brain analysis of generosity

**Fig. 8:**
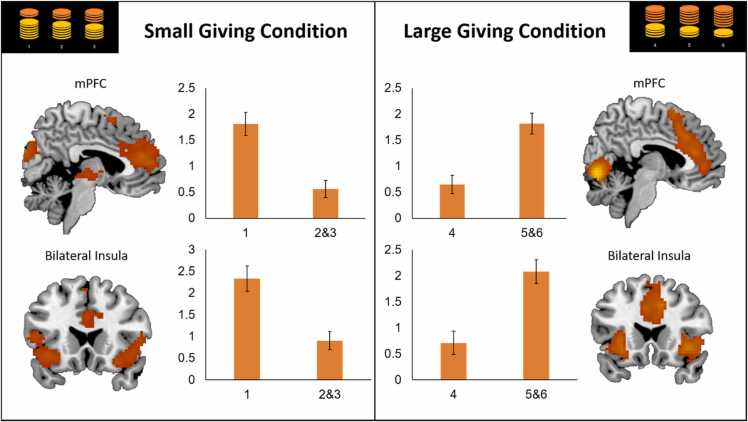
Increased activation in the mPFC and bilateral insula for minimal vs. generous giving (i.e., giving 1 vs. 2 or 3 coins) in the small giving condition, and for generous vs. minimal giving (i.e., giving 5 or 6 coins) in the large giving condition.

To examine neural activation associated with relative generosity within the small and large giving conditions, we examined a whole brain contrast of ‘minimal vs. generous giving’ and ‘generous vs. minimal giving’ within these conditions. The contrast ‘minimal vs. generous giving’ in the small giving condition and the reverse contrast ‘generous vs. minimal giving’ in the large giving condition both resulted in increased activation in mPFC and bilateral AI, see Fig. 8 and Table 6. No significant linear or quadratic age effects were found.

**Table 6:** MNI coordinates of local maxima activated for the t-tests minimal > generous giving in the small giving condition and generous > minimal giving in the large giving condition. Results were calculated using a primary voxel-wise threshold of p < .001 (uncorrected), with a cluster corrected threshold of *p* < .05 FDR corrected.

Area of activation	MNI Coordinates			Test statistic	Cluster Size
	x	y	z	*F/t*	
*T-test Minimal > Generous Giving In Small Giving Condition (FDRc < 0.001 = 59)*					
Left Lingual	-9	-79	-5	14.31	840
Right Anterior Cingulum (i.e., mPFC)	6	44	19	5.54	802
Left Insula	-36	20	-11	4.56	264
Right Frontal Inferior Orbital	36	26	-14	5.04	242
Right Temporal Middle	51	-31	-5	4.72	224
Left Temporal Middle	-63	-49	-2	4.44	195
Right Insula	33	-28	22	4.60	198
Right Thalamus	6	-19	-2	4.21	130
Left Supplementary Motor Area	-6	5	61	4.72	99
Right Temporal Middle	48	5	-29	4.84	59
*T-test Generous* vs. *Minimal Giving In Large Giving Condition (FDRc < 0.001 = 75)*					
Right Middle Cingulum (i.e., mPFC)	12	20	37	6.45	1180
Right Lingual	12	-76	-11	13.05	632
Right Insula	39	17	-5	7.37	281
Left Insula	-30	26	-2	6.35	218
Right Frontal Inferior Operculum	36	14	28	4.32	100
Left Inferior Occipital	-45	-79	-5	5.45	96
Right Frontal Inferior Triangularis	45	38	13	4.74	75

Note: Names were based on the aal toolbox in SPM. For functional regions discussed throughout the paper, both the aal label and functional label (between brackets) are displayed. See https://neurovault.org/collections/ICTLVEUU/ for a full, unthresholded overview of activation.

## 4 Discussion

This study examined three aspects of giving behavior in 9–19-year-old adolescents. The first aim was to examine behavioral and neural profiles of giving in small (low personal cost) versus large size (high personal cost) giving conditions. The second aim was to examine whether these behaviors and neural patterns were dependent on whether the target was a friend or unfamiliar peer. The third aim tested giving that was being observed by peers relative to giving anonymously. Behaviorally, adolescents gave more in the small than in the large giving condition, suggesting that participants were more generous when personal costs were relatively low. Moreover, especially in the small size giving condition, adolescents gave more to friends than to strangers, and gave more when observed by peers. With respect to age differences, we found that older participants differentiated more between giving to friends and unfamiliar others, specifically in the small size giving condition. These findings are consistent with our pre-registered hypotheses 1–2 and with prior studies showing a developmental increase in target differentiation when giving to others in adolescence (Güroğlu et al., 2014, Schreuders et al., 2019). The target differentiation was specific to the small giving condition in our study. This could reflect biased giving rates towards friends for small (low personal cost) giving, but also more equitable giving towards friends and unfamiliar peers for large (high personal cost) giving. This suggests that the magnitude of giving could be an important factor for minimizing group biases (Do and Telzer, 2019, Hackel et al., 2017). Future studies should aim to further unravel how different dimensions of giving magnitudes (e.g., costliness and level of inequity) interact in shaping giving decisions towards in- and out-group targets.

Our observation of higher giving in the audience compared to anonymous condition, specifically in the small giving condition, is consistent with a prior study in adults (van de Groep et al., 2020b) and in adolescents (Van Hoorn et al., 2016) and confirms hypothesis 3. Next, we observed longer reaction times for giving to a friend versus unfamiliar peer (specific to the large giving condition), and with the audience present. One interpretation for these effects is that adolescents give more deliberately in the friend and peer presence conditions due to concerns for their future reputation, as these conditions were also associated with higher levels of giving. Furthermore, our results suggest that older adolescents make faster giving decisions in the small compared to large giving condition when an audience was present, suggesting that such giving decisions are easier to make for older compared to younger adolescents. Together, the findings fit with a larger body of studies showing that adolescents’ prosocial behaviors are influenced by social contextual factors (Güroğlu, 2020, Telzer et al., 2015, van den Bos et al., 2012, van den Bos et al., 2018, van Hoorn et al., 2016).

### 4.1 Neural correlates of small versus large size giving

The next aim of this study was to examine the neural correlates of giving in adolescence with a task design optimized for comparing giving small (low costly) versus large (high costly) amounts. Consistent with a prior study in adults using the same paradigm (van de Groep et al., 2020b), and with pre-registered hypothesis 5, we found that adolescents showed stronger activity in a pre-defined mPFC ROI in the small versus large giving condition, which was confirmed by the whole brain analyses. This finding fits with a larger set of neuroimaging studies focused on giving decisions in adults showing involvement of the mPFC, a region which often co-activates with the superior temporal sulcus, ventral striatum, AI, and TPJ, and IPL (Blakemore, 2008, Cutler and Campbell-Meiklejohn, 2019, Güroğlu et al., 2014, Telzer et al., 2015, van de Groep et al., 2020b). However, here were observed no activation for small versus large size giving in the following pre-defined ROIS: the nAcc, dACC,TPJ, and LPFC.

Next, we performed exploratory whole brain analyses to examine whether the neural signature of giving was influenced by participants’ generosity within the small and large giving condition. The mPFC and AI showed dependency on giving decisions within the small and large giving conditions, similar to previous findings in adults (van de Groep et al., 2020b). Both the mPFC and AI correlated with the most extreme choices, including minimal giving in the small giving condition (relative to generous giving), and generous giving in the large giving condition (relative to minimal giving; partly confirming hypothesis 5). These findings provide evidence for a role of the mPFC and AI in detecting norm violations or saliency (Cutler and Campbell-Meiklejohn, 2019, Güroğlu et al., 2009, Güroğlu et al., 2014, van de Groep et al., 2020b). It should be noted that in adults, these regions were most strongly activated for generous giving independent of giving magnitude context (van de Groep et al., 2020b). Combined, these findings could reflect a developmental shift from multiple-context saliency detection in adolescents (i.e., minimal giving and generosity) to saliency detection for generosity in adulthood. Future studies should follow-up this hypothesis using longitudinal assessments to examine within-person changes, and novel designs to separate contextual restrictions on giving (small versus large giving conditions) with participants’ actual giving preferences (e.g., minimal or generous giving).

To address developmental effects, we examined age differences in pre-defined ROIs. Contrary to our expectations, none of these ROI analyses resulted in developmental patterns. However, exploratory whole brain analyses revealed an age-related increase in lateral and anterior PFC activation for giving small (low-costly) versus large (high-costly) amounts. Inspection of the developmental pattern shows a shift from more lateral/anterior PFC activity in the large giving condition early in adolescence, to the small giving condition later in adolescence. Developmental increases in LPFC activation have been well documented in prior studies on cognitive control (Crone and Steinbeis, 2017), receiving trust (van den Bos et al., 2011), and emotion regulation development (Silvers and Guassi Moreira, 2019). Possibly, the lateral PFC plays an important regulatory role in balancing needs for self and others; for instance by inhibiting impulses or integrating norms into decisions (Cutler and Campbell-Meiklejohn, 2019). It should be noted that the pre-registered ROIs also included a dlPFC region, but the whole brain contrasts showed that developmental differences were most pronounced in a different area of the prefrontal cortex. Apart from the dlPFC, this study showed other discrepancies between ROI and whole brain analyses. This includes no ROI peer presence effects, but whole brain effects in the fusiform and lingual gyrus; and no ROI target effects except for the mPFC, whereas whole brain analyses revealed differences in several regions of interest, such as the TPJ-IPL and lateral prefrontal cortex. These discrepancies have several broader implications. First, it shows the importance of carefully selecting ROIs. ROIs that are based on a synthesis of prior research (i.e., are created through Neurosynth) may be less anatomically and functionally demarcated than ROIs that are based on one specific study that measures the exact construct of interest. Second, it shows that even though pre-registration is useful to formulate a priori hypotheses, exploratory analyses are an important additional way to capture meaningful neural activation that might otherwise be overlooked, especially for novel experimental paradigms. This can result in interesting new hypotheses for future research. Third, it highlights the importance of performing neuroimaging research in developmental samples, such that ROIs are not only applicable to adult neural activation, but also to developing neural activation patterns. Fourth, it highlights that the field of developmental cognitive neuroscience would benefit from a consensus on how to weigh a priori, confirmatory ROI analyses versus whole brain exploratory analyses. ROI analyses boost statistical power and therefore lead to more robust results, but are only valid when the ROIs are selected carefully and appropriately for the sample of interest. Whole brain exploratory analyses can identify neural activation that was not hypothesized but are marked by decreased spatial certainty and resolution because of normalization and spatial smoothing (Szycik et al., 2009). In short, both ROI and whole brain analyses have strengths and weaknesses, which is why it is beneficial to combine both approaches in one study.

The current experimental design was optimized for comparing small versus large giving, and therefore differed from prior operationalizations of the Dictator Game, the economic game traditionally used to study giving. Most prior neuroimaging studies, the majority of which were performed in adults, used operationalizations where the participant could 1) either reject or accept a pre-defined donation, or 2) choose between two proposed pay-offs (Cutler and Campbell-Meiklejohn, 2019, Telzer et al., 2015). In these studies, relatively generous choices were associated with activation in the ventral striatum, mPFC and LPFC, especially when made voluntarily (Cutler and Campbell-Meiklejohn, 2019, Harbaugh et al., 2007, Hare et al., 2010). The current paradigm adds to this literature by showing that mPFC and AI were activated for both minimal versus generous giving in adolescents, possibly reflecting either feelings of generosity (Cutler and Campbell-Meiklejohn, 2019) or saliency detection (Güroğlu, Will et al., 2014). Overall, the current literature would benefit from developmental studies with experimental designs, which explicitly consider factors such as saliency, voluntarity, generalizability, and the presence of extrinsic rewards.

### 4.2 Neural correlates of familiarity and audience effects

The second and third aim of this study were to test whether neural correlates of giving considerations were dependent on whether the target was a friend or unfamiliar peer and whether participants were observed by an audience or decisions were anonymous. From all pre-registered ROIs, the AI was the only region showing sensitivity to these social contextual manipulations. Specifically, the AI showed stronger activity when giving to friends compared to unfamiliar peers, but only in the audience condition, whereas activation between friends and unfamiliar peers did not differ in the anonymous condition. These findings possibly reveal that the audience condition amplifies social concern or saliency for friends (van Hoorn et al., 2019). Across adolescent development, the peer context becomes an important additional social consideration. For example, a prior developmental study showed that adolescents increasingly consider friends’ outcomes in their decision making throughout adolescence and into young adulthood (Powers et al., 2018). There is by now accumulating evidence that the peer context is associated with increased reward sensitivity at the neural level, which is related to both risky and prosocial behaviors (Chein et al., 2011, Do et al., 2017, Schreuders et al., 2019, Smith et al., 2018, Telzer et al., 2010, van Hoorn et al., 2016). Adolescents’ orientation and motivation to be accepted by and gain status among their peers drive many goal-directed behaviors, resulting in increased saliency in situations where this can be achieved (van Hoorn, Fuligni et al., 2016).

Contrary to predictions, no target or peer presence effects were observed in the mPFC ROI (Van Hoorn et al., 2016). This finding seemingly contradicts a plethora of studies suggesting stronger mPFC activity in adolescents in social fMRI tasks which require mentalizing, including assessments of reading the mind in the eyes (Gunther Moor et al., 2012), social perspective taking (Dumontheil et al., 2010), and reciprocity (Crone and Fuligni, 2020, van den Bos et al., 2011). Possibly, the social manipulations used in this study put less demands on mentalizing compared to other studies. For example, prior studies have shown that peer monitoring and influence have larger effects on both risk taking (Powers et al., 2018, Somerville et al., 2019) and giving behavior (van Hoorn, Dijk et al., 2016) than peer presence per se, and this might require different levels of mentalizing. To further explore this possibility, future studies should examine social influence effects using more diversity in peer influences.

A second unexpected finding was the absence of developmental effects in the a priori defined TPJ. Prior studies have revealed TPJ activation in response to peer presence during giving (Van Hoorn et al., 2016), and trust and reciprocity decisions (Fett et al., 2014, van den Bos et al., 2011), suggesting that the TPJ is an important region for taking the perspective of others and moderating behavioral choices (Carter and Huettel, 2013, Crone and Fuligni, 2020, Fett et al., 2014, van den Bos et al., 2011, Van Hoorn et al., 2016). In the current study, exploratory whole brain analyses demonstrated stronger anterior IPL/TPJ activity when giving to friends than to unfamiliar peers, consistent with a prior study in adults (van de Groep et al., 2020b). The TPJ and IPL are congeneric parietal regions comprising of sub regions that fulfill different roles in a global function of mentalizing, integrating social contexts, and thinking about self and others (Carter and Huettel, 2013, Schurz et al., 2014, van de Groep et al., 2020b). However, despite prior studies that show increased activation in the TPJ with increasing age for fair decisions (Güroğlu et al., 2011, van den Bos et al., 2011), in the current study we observed no age effects in this region, possibly because the age range was relatively narrow to detect developmental differences.

The exploratory whole brain analyses revealed additional activation for giving to friends versus unfamiliar peers in the SMA and dlPFC, regions often implicated in cognitive control (Cole and Schneider, 2007) and positive affect, including peer acceptance (Achterberg et al., 2018). Prior studies have implicated the dlPFC and TPJ, which both showed sensitivity to target in this study, as possible neural facilitators of perspective taking (Yang et al., 2020). It is an intriguing question for future research whether perspective taking can be trained, and whether this reduces in- versus out-group differentiation.

We also addressed possible interactions between giving and social contextual factors. In whole brain analyses we observed target x giving condition dependent activation in the precuneus, a region involved in self- versus other-referential processing and perspective taking (Cavanna and Trimble, 2006, Cutler and Campbell-Meiklejohn, 2019). Specifically, activation was higher for the friend in the large giving condition, and higher for the unfamiliar peer in the small giving condition, which was accompanied by longer reaction times in these conditions. Prior studies have shown that the precuneus is strongly activated in situations where giving is relatively high-costly in terms of monetary or reputational outcomes, which might require additional deliberation (Do et al., 2019, van der Meulen et al., 2018, van der Meulen et al., 2018). This would fit with a potential role of the precuneus in perspective taking (Cutler and Campbell-Meiklejohn, 2019).

### 4.3 Limitations and future directions

This study had several limitations that should be addressed in future research. The first limitation is that the current study cross-sectionally examined age-related differences in generosity. The data described here represents the first measurement wave of the longitudinal Brainlinks study. In the future, the longitudinal within-subjects examinations of the development of giving and other prosocial behaviors acquired within this study can be used to address this limitation. Nonetheless, the current findings, combined with other studies, still inform why some contextual considerations (i.e., personal costs, target, peer presence) and brain systems are more or less influential on the development of giving behavior. A first important take-away is that there is converging evidence for a developmental increase in giving in adolescence specifically for the friend target, which underlines the importance of friends in adolescence. Studies that examined more general in- versus out-group giving (Do et al., 2019) or giving to different types of non-friend peers (Güroğlu et al., 2014) in adolescents observed no developmental increases. Here, we observed increased giving to friends relative to unfamiliar others in the context of small size giving, which is consistent with other studies in adolescents (Güroğlu et al., 2014). Importantly, the developmental sensitivity for giving to friends may be specific to mid-to-late adolescence, as earlier work found no developmental changes in giving to friends in 8–12-year-olds (Asscheman et al., 2020). Adolescents’ inclination for giving to friends is also evident in our neuroimaging findings. Here, we observed that the TPJ, SMA, dlPFC, and right insula were more sensitive to the friend versus unfamiliar target, while earlier studies in adults only reported involvement of the TPJ (van de Groep et al., 2020b). This suggests that additive emotional, saliency, and cognitive control processes may be involved for giving to a friend in adolescents compared to young adults. A second important take-away is that neural age-related differences were mainly observed in cortical, but not sub-cortical regions. This might reflect adolescents’ tendency to use more cognitive-mentalizing strategies when performing social tasks as they grow older (Crone and Fuligni, 2020), which is consistent with evidence from prior neuroimaging studies that examined strategic giving (Güroğlu et al., 2009; 2011; Steinbeis et al., 2012; van den Bos et al., 2011). In these studies, increased recruitment of the TPJ and prefrontal regions were indicative of perspective taking and cognitive control. Together, this suggests that activation in cortical neural regions contributes to developmental changes and increased sophistication in giving across adolescence (Crone and Fuligni, 2020). These suggestions should be addressed in future longitudinal studies with a within-subject design. A second limitation of this study is that it only focused on two targets of giving: a friend and unfamiliar peer. While peer targets are important in adolescence (van Hoorn, Fuligni et al., 2016), our understanding of the neural and behavioral correlates of giving would benefit from including both peer- and non-peer targets (e.g., parents, siblings, acquaintances) with various degrees of familiarity in future studies. Third, the current sample was recruited through local and online advertisements, thereby possibly limiting the diversity of the sample. Future studies should test whether the current results replicate in more diverse samples (e.g., in terms of gender and ethnicity) and should try to reach underrepresented groups of adolescents. Fourth, the peer audience in our study was prerecorded and provided ratings of participants’ behavior later in time, whereas other studies have used real-time audiences (Van Hoorn et al., 2016; Somerville, 2013; Izuma et al., 2010). The lack of ROI results and unexpected whole brain results related to the audience condition may stem from the delayed timing or lower salience of our peer presence manipulation. Finally, future studies should include emerging young adults in addition to adolescents to fully understand changes related to the transition from adolescence into adulthood (Willoughby et al., 2014).

## 5 Conclusion

This study examined important contextual influences on giving behavior that have been understudied in prior studies in adolescents: giving magnitude (small versus large size giving), target (giving to a friend versus unfamiliar other), and peer presence (giving with an audience or anonymously), as well as possible differences across ages 9–19. We demonstrated that giving very small or very large amounts was associated with increased activity in the mPFC and AI. Furthermore, we demonstrated an age-related increase in lateral and anterior PFC activation across adolescent development for giving small versus large amounts, showing a developmental shift from stronger activity for large (high-costly) giving to small (low-costly) giving. Finally, with increasing age adolescents gave more to friends and less to unfamiliar peers, especially in the small giving context. Exploratory whole brain analyses revealed no overlap in brain regions pertaining to the target and peer presence contexts, suggesting that contextual factors have partly interactive and partly additive effects on giving. These results shed new light on the neural networks involved in balancing the needs of self and others, and highlight increasing in-group (friend) versus out-group differentiation in adolescence.

## Data statement

Research Data will be uploaded to Erasmus University Rotterdam’s Data Repository upon acceptance. At that point, access can be requested from the study’s authors.

## Declaration of Competing Interest

The authors declare that they have no known competing financial interests or personal relationships that could have appeared to influence the work reported in this paper.

## Data Availability

Data will be made available on request.
