# Supplementary material for: Growing in generosity? The effects of giving magnitude, target, and audience on the neural signature of giving in adolescence
Source: Dev Cogn Neurosci. 2022 Feb 9;54:101084. doi: 10.1016/j.dcn.2022.101084 (PMC8857499; doi:10.1016/j.dcn.2022.101084)
Supplement: Supplementary file 1 — Supplementary material [file mmc1.docx]

| *Measures* | *Average values* |
| --- | --- |
| Means (SD) | 0.079 (0.08) |
| Minimum | 0.0016 |
| Maximum | 2.9748 |
| Number of small spikes (.9-3mm) | 1.45 |
|  | *Number* |
| Participants with 0 spikes (.9-3mm) | 93 |
| Participants with small spikes (.9-3mm) | 35 |

Supplement 1

**Supplementary Table 1:** Movement information after exclusion of participants with >.3mm movement


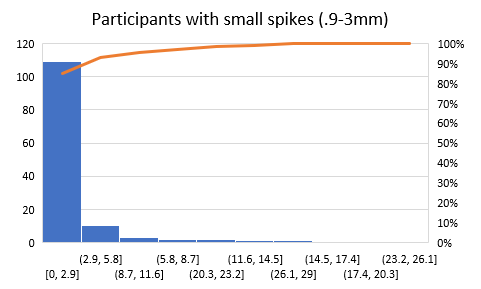


**Supplementary Figure 1:** Number of participants with small spikes (.9-3mm). The x-axis shows categories reflecting the number of small spikes. The y-axis reflects the number of participants within each category.

| PPN | Mean | stdev | min | max | Nr. of spikes (i.e., > .9mm) |
| --- | --- | --- | --- | --- | --- |
| 1 | 0.093836 | 0.169465 | 0.005728 | 2.084355 | 4 |
| 2 | 0.153558 | 0.156385 | 0.006756 | 0.973543 | 2 |
| 3 | 0.095539 | 0.165684 | 0.004561 | 2.01902 | 3 |
| 4 | 0.055123 | 0.057882 | 0.003254 | 1.04213 | 1 |
| 5 | 0.052653 | 0.060299 | 0.005508 | 1.106317 | 1 |
| 6 | 0.185345 | 0.237073 | 0.011029 | 1.712693 | 11 |
| 7 | 0.082127 | 0.086494 | 0.004915 | 0.948127 | 1 |
| 8 | 0.1219 | 0.101774 | 0.008922 | 1.007052 | 1 |
| 9 | 0.099774 | 0.165717 | 0.003516 | 1.808145 | 4 |
| 10 | 0.074266 | 0.109193 | 0.004998 | 1.509983 | 1 |
| 11 | 0.07251 | 0.111467 | 0.004416 | 2.040812 | 1 |
| 12 | 0.065756 | 0.124468 | 0.002655 | 1.462896 | 4 |
| 13 | 0.103581 | 0.112521 | 0.004087 | 0.979952 | 1 |
| 14 | 0.147065 | 0.247692 | 0.003294 | 1.870332 | 11 |
| 15 | 0.094579 | 0.111122 | 0.008152 | 1.113625 | 2 |
| 16 | 0.066701 | 0.106514 | 0.003842 | 1.849223 | 1 |
| 17 | 0.107374 | 0.108724 | 0.005205 | 1.118187 | 1 |
| 18 | 0.169952 | 0.223783 | 0.004185 | 1.601298 | 10 |
| 19 | 0.173537 | 0.293683 | 0.004407 | 2.442657 | 12 |
| 20 | 0.104297 | 0.139667 | 0.002299 | 1.563069 | 3 |
| 21 | 0.082779 | 0.13113 | 0.003988 | 1.69999 | 3 |
| 22 | 0.232859 | 0.376235 | 0.003847 | 2.837462 | 23 |
| 23 | 0.118377 | 0.115295 | 0.010597 | 1.442318 | 1 |
| 24 | 0.101358 | 0.155197 | 0.006987 | 1.879987 | 3 |
| 25 | 0.148449 | 0.238373 | 0.007738 | 2.974833 | 8 |
| 26 | 0.084402 | 0.152705 | 0.005379 | 2.121363 | 2 |
| 27 | 0.107932 | 0.100126 | 0.010657 | 1.298366 | 1 |
| 28 | 0.103214 | 0.202594 | 0.004533 | 2.187229 | 5 |
| 29 | 0.12941 | 0.154773 | 0.009193 | 1.467264 | 4 |
| 30 | 0.177966 | 0.191942 | 0.007661 | 1.496194 | 7 |
| 31 | 0.099553 | 0.103375 | 0.001931 | 0.994603 | 1 |
| 32 | 0.227127 | 0.303294 | 0.012977 | 1.760259 | 21 |
| 33 | 0.092284 | 0.133121 | 0.00604 | 1.026428 | 3 |
| 34 | 0.324148 | 0.399072 | 0.00885 | 2.955048 | 27 |
| 35 | 0.070318 | 0.083236 | 0.002923 | 1.101038 | 1 |

**Supplementary Table 2.** Specific movement information for the 35 participants who displayed small motion spikes (.9-3mm).

**Data access**

Raw data for each participant can be provided by the first author upon request.
