# Supplementary material for: Growing in generosity? The effects of giving magnitude, target, and audience on the neural signature of giving in adolescence
Source: Dev Cogn Neurosci. 2022 Feb 9;54:101084. doi: 10.1016/j.dcn.2022.101084 (PMC8857499; doi:10.1016/j.dcn.2022.101084)
Supplement: Supplementary file 2 [file mmc2.docx]

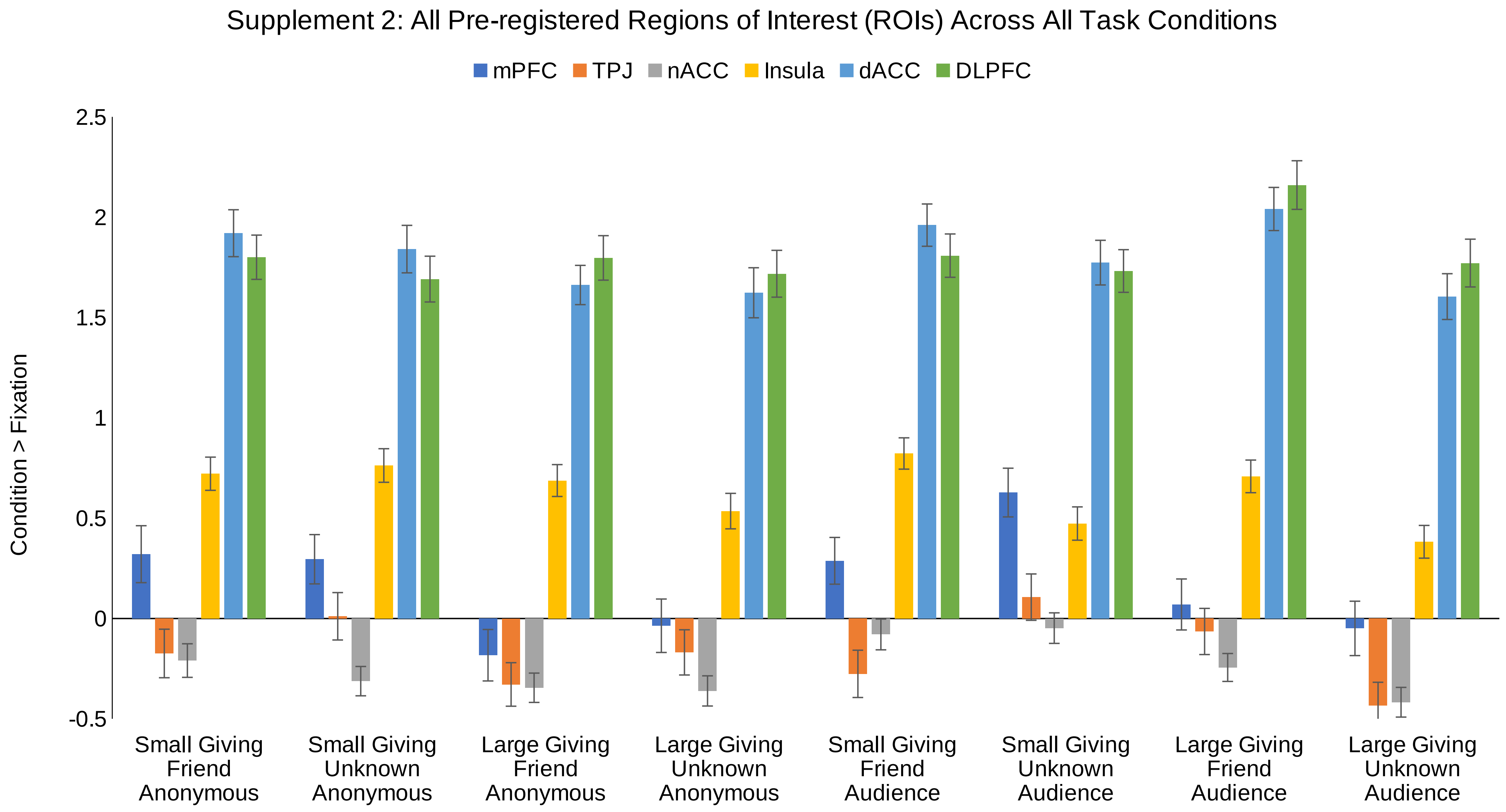

Supplement 2: All Pre-registered Regions of Interest (ROIs) Across All Task Conditions
mPFC
TPJ
nACC
Insula
dACC
DLPFC
Condition > Fixation
2.5
2
1.5
1
0.5
0
-0.5
Small Giving Friend Anonymous
Small Giving Unknown Anonymous
Large Giving Friend Anonymous
Large Giving Unknown Anonymous
Small Giving Friend Audience
Small Giving Unknown Audience
Large Giving Friend Audience
Large Giving Unknown Audience
